# Antibiotic Resistance Pattern of Pathogens Isolated from Pediatric Patients during and after the COVID-19 Pandemic

**DOI:** 10.3390/antibiotics13100966

**Published:** 2024-10-13

**Authors:** Andreea-Loredana Golli, Simona Georgiana Popa, Monica Laura Cara, George-Alin Stoica, Dragos Fortofoiu, Maria Stoica

**Affiliations:** 1 Department of Public Health and Management, University of Medicine and Pharmacy of Craiova, 200349 Craiova, Romania; andreea.golli@umfcv.ro (A.-L.G.); monica.cara@umfcv.ro (M.L.C.); 2 Department of Diabetes, Nutrition and Metabolic Diseases, University of Medicine and Pharmacy of Craiova, 200349 Craiova, Romania; 3 Department of Pediatric Surgery and Orthopedics, University of Medicine and Pharmacy of Craiova, 200349 Craiova, Romania; alin.stoica@umfcv.ro; 4 Department of Internal Medicine, University of Medicine and Pharmacy of Craiova, 200349 Craiova, Romania; fortofoiudragos@gmail.com; 5 Department of Intensive Care, University of Medicine and Pharmacy of Craiova, 200349 Craiova, Romania; maria.stoica@umfcv.ro

**Keywords:** pathogens, children, antimicrobial resistance, COVID-19, pandemic

## Abstract

**Background/Objectives**: The present study aims to highlight the possible significant changes due to the COVID-19 pandemic in the resistance of pathogens involved in cases of pediatric infections. **Methods**: This study included children hospitalized in the Pediatric Intensive Care Unit, Surgery and Pediatrics from a tertiary teaching hospital, during and after the COVID-19 period (2020–2023). **Results**: The research included 845 samples collected during 2020–2023, from 685 pediatric patients. A total of 937 bacterial isolates were obtained, of which 509 isolates (54.32%) were Gram-negative bacteria. Around 30% of all the pathogens were multidrug-resistant (MDR), with a statistically significant increase post-pandemic, in the case of the MDR *Escherichia coli* strains (*p* < 0.05). A very high percentage of MDR *Acinetobacter* spp. isolates was found, with an important, but not statistically significant, increase in the post-pandemic period. The highest percentage of the MDR Gram-positive pathogens was registered in the case of *S. aureus* strains (31.80%). Over 20% of the *Coagulase-negative Staphylococci (CoNS)* strains isolated between 2020 and 2023 were MDR, with an important increase in the post-COVID-19 period. The proportion of carbapenem-resistant Gram-negative pathogens significantly decreased in the post-COVID-19 period compared with the COVID-19 period (*p* < 0.05), especially in the case of the *Klebsiella* spp. strains. **Conclusions**: Our findings revealed the increase in the post-COVID-19 period of the prevalence of MDR strains of *Acinetobacter* spp., *CoNS*, and *Escherichia coli* isolated in pediatric patient samples and a significant decline in the trend of the carbapenem-resistant Gram-negative pathogens, which may be due to the testing rate and to the specific pathology of the pediatric patients hospitalized in the two periods.

## 1. Introduction

Antimicrobial resistance (AMR) was identified in 2022 by the Council of the European Union as one of the top three priority health threats facing humanity [1]. The estimated number of people who die every year in the EU/EEA as a direct consequence of an infection due to bacteria resistant to antibiotics is over 35,000 [1] and, by 2050, the number of deaths is expected to rise to 10 million deaths every year [2]. In addition to increasing the risk of death and disability, AMR has a negative financial impact on both patients and their families, as well as on health systems, due to the extension of the duration of hospitalization and the increase in the cost of health care.

The COVID-19 pandemic was a global threat that put pressure on all health services in EU/EEA countries, with possible negative consequences on antimicrobial resistance in the long term, due to the improper use of antibiotics, which is the main driver in the development of drug-resistant pathogens, involved in healthcare-associated infections.

According to ECDC, the latest data showed significantly increasing trends in the number of infections and attributable deaths for almost all bacterium–antibiotic resistance combinations, especially in healthcare settings [3]. Thus, there was a more than twofold increase in the number of reported cases of *Acinetobacter* species resistant to different antimicrobial groups than the average recorded in the pre-pandemic period (2018–2019). Additionally, the percentage of *Klebsiella pneumoniae* cases resistant to carbapenems increased by more than 30% in 2020 and by another 20% in 2021 [3]. A favorable therapeutic result is difficult to obtain in cases of infections with this etiology.

The lowest AMR percentages were reported by countries in the north of Europe, and the highest by countries in the south and east of Europe. These countries also reported the highest percentages and estimated incidence of bloodstream infections with resistant bacteria in 2022 [4].

The results of the ECDC point prevalence survey (PPS) of healthcare-associated infections (HAIs) and antimicrobial use in European acute care hospitals (2022–2023), including from Romania, highlighted the increase in the percentage of the *Klebsiella* species among the five most frequently isolated microorganisms in HAIs, continuing to reflect the ongoing epidemic of carbapenem-resistant Gram-negative bacteria in Europe [4].

Romania is one of the Eastern European countries with a high incidence of multidrug-resistant pathogens. According to the existing data on invasive isolates available from the European Antimicrobial Resistance Surveillance Network (EARS-Net), in 2019, Romania recorded the highest incidence in the EU of methicillin-resistant *Staphylococcus aureus* (MRSA) bloodstream infections (13.7/100,000) [1], which are among the leading health-care-associated and community-acquired infections worldwide [5]. The incidence of carbapenem-resistant *Klebsiella pneumoniae* bloodstream infections placed Romania in third place among EU countries in 2019 (7.12/100,000), reflecting the high burden of MDR pathogens in this country [1]. Both pathogens, together with MDR-TB (multidrug-resistant tuberculosis), which is also a public health problem in Romania [6], are on the 2024 WHO Bacterial Priority Pathogens List, which updates and refines the prioritization of antibiotic-resistant bacterial pathogens to address the evolving challenges of antibiotic resistance [7].

AMR raises great concern in the case of the pediatric population due to limited treatment options for MDR pediatric infections, which accounted for up to 30% of all cases in Europe [8]. Around 200,000 of the deaths caused by multidrug-resistant bacteria occur in newborns [9].

Since most studies have targeted AMR in adults, there are few available data about the impact of AMR and the consequences of infection with multidrug-resistant pathogens on children.

Globally, about 20% of the 1.27 million estimated deaths directly attributable to AMR in 2019 occurred among children under the age of 5, with more than 99 percent being from low- and middle-income countries [10].

According to a report on the global burden of bacterial antimicrobial resistance (Global burden of bacterial antimicrobial resistance 1990–2021: a systematic analysis with forecasts to 2050. Naghavi, Mohsen et al., *The Lancet*, Volume 404, Issue 10459, 1199–1226), almost 200,000 deaths attributable to AMR globally were recorded in 2021 in children younger than 5 years. *Klebsiella pneumoniae*, *Streptococcus pneumoniae*, and *E. coli* were the most frequent pathogens involved in these deaths [11].

In order to increase the awareness of medical staff and the population regarding MDR infections in the post-pandemic context, especially among children, studies are needed to assess the impact of the COVID-19 pandemic on AMR.

In the current research, we investigated the trend of the antimicrobial resistance pattern of pathogens isolated in samples collected from pediatric patients hospitalized in a tertiary teaching hospital from Romania during and after the COVID-19 pandemic (2020–2023).

## 2. Results

### 2.1. Distribution of the Main Isolates

This research included 845 samples collected during 2020–2023 from 685 pediatric patients admitted in the Pediatric Intensive Care Unit (PICU) (134—19.56%), Surgery ward (191—27.88%), and Pediatrics (360—52.56%). Their average age was 6 ± 5.82 years, and 54.89% (376) were males.

During the study period, most samples came from pus/wound swabs (237—28.05%), the respiratory tract (231—27.34%), and urine (205—24.26%). More than half of the samples were collected during the COVID-19 pandemic (2020–2021), followed by a lowering trend in 2022 and a new increase in 2023, after the end of the epidemiological alert period (Table 1).

**Figure 1:**
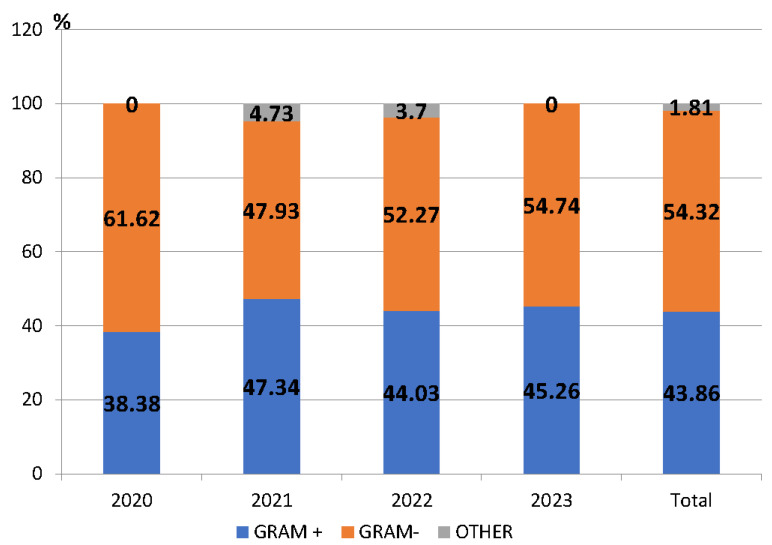
Proportion of Gram-positive and Gram-negative pathogens among the isolated pathogens, County Emergency Clinical Hospital Craiova, Romania, 2020–2023.

A total of 937 bacterial isolates were obtained, excluding cases where it was more than one isolate of the same pathogen from the same patient and the same site of infection. Among these, 509 isolates (54.32%) were Gram-negative bacteria, 411 (43.86%) were Gram-positive, and 17 (1.81%) were other bacterial species (Figure 1).

The number of isolated bacteria was different from year to year. The highest percentage of isolated microorganisms between 2020 and 2023 was found in 2023 (327/937—34.90%), and this was significantly higher than in 2022 (243/937—25.93%) (*p* < 0.05).

During the entire study period, Gram-negative pathogens predominated. Of these, the most frequently detected were *Escherichia coli* (36.15%), *Klebsiella* species (spp.) (26.52%), *Pseudomonas* spp. (17.09%) and *Acinetobacter* spp. (7.46%). The percentage of Gram-negative bacteria significantly decreased in 2021 compared to the previous year (*p* < 0.05) (Figure 1). The most frequently detected Gram-positive pathogens were *Staphylococcus aureus (S. aureus)* (58.15%), *Enterococcus* spp. (15.57%), and *Streptococcus pneumoniae* (13.38%) (Table S1).

### 2.2. Antimicrobial Resistance in Main Baterial Species

#### 2.2.1. Gram-Positive Pathogens

*Staphylococcus aureus* was the most frequently isolated microorganism, representing 25.51% of all pathogens. Most commonly, it was isolated from pus/wound swabs (34.31%) and the respiratory tract (27.62%) (Table S2). Two-thirds of the strains (151/239) were detected in the post-COVID-19 period (2022–2023) (Table 2).

During the COVID-19 period, it was most commonly isolated from the respiratory tract (37/88) and pus/wound swabs (30/88). In the post-COVID-19 era, *S. aureus* was found mainly in the nose/pharynx (50/151) and in pus/wound swabs (52/151). The highest resistance was recorded to penicillin (80.17%,) erytromycin (75.53%), clarithromycin (65.66%), and clindamycin (68.51%). Almost 50% of all the tested strains (114/175) were resistant to oxacillin. Regarding the distribution of resistant oxacillin strains by year, the highest percentage was recorded in 2022 (66.17%—45/68). The vancomycin resistance rate was under 10% and no strains resistant to linezolid were found (Table 3).

*Enterococcus s*pp. was mainly isolated from urine (40.63%) and pus/wound swabs (28.12%) (Table S2). More than half of the *Enterococcus* spp. isolates were resistant to ciprofloxacin and penicillin, and a third to levofloxacin. The lowest resistance was found in the cases of vancomycin (8.33%) and linezolid (3.7%) (Table 3). Two-thirds of the strains were detected in the post-COVID-19 period (Table 2).

Almost 80% of the tested strains of *Streptococcus pneumoniae* were resistant to penicillin, while all strains were sensitive to vancomycin, linezolid, and rifampicin (Table 3). The percentage of the strains was almost equal in the two analyzed periods (Table 2).

#### 2.2.2. Gram-Negative Pathogens

*Escherichia coli* was the most frequently isolated bacterium, with a proportion of 19.64%. More than 50% was isolated from urine (94/184), followed by pus/wound swabs (64/184) (Table S2). The percentage of isolated strains was higher in the post-COVID-19 period (63.58%) (Table 2). The highest resistance rate was identified for amoxicillin/clavulanic acid (62.11%) and second-generation cephalosporins (cefuroxime—61.76%), and the lowest to carbapenems (imipenem—5.03%, meropenem—4.39%). Almost 30% of the *Escherichia coli* strains were resistant to colistin (Table 4). A share of 33.15% (61/184) of the strains were MDR, and the carbapenem resistance rate was 4.89% (9/184).

Approximately 60% of *Klebsiella* spp. strains were isolated in the post-COVID-19 period. Over the entire period analyzed (2020–2023), almost half of the *Klebsiella* spp. strains were isolated from urine (62/135) and around 20% (28/135) from the respiratory tract (Table S2).

More than 50% of the *Klebsiella* spp. strains were resistant to first-, second-, third-, and fourth-generation cephalosporins: cefuroxime (73.13%), cefazolin (70.88%), ceftazidime (60.83%), ceftriaxone (59.65%), cefepime (52.7%), piperacillin/tazobactam (58.11%), and gentamicin (54.64%). Around 20% of the strains were carbapenem-resistant and approximately 25% were resistant to colistin (Table 4). Almost half of the strains were MDR (45.18%) and 22.96% (31/135) were carbapenem-resistant.

In the case of *Pseudomonas* spp. strains, the percentage of the isolated strains was higher in the post-COVID-19 period (56.32%) (Table 2). Most of the strains were identified in the respiratory tract (43.68%), followed by pus/wound swabs (24.14%). A very high level of antimicrobial resistance was revealed against the first- generation (cefazolin—80%) and third-generation cephalosporins (ceftriaxone—71.43%; ceftazidime—60.83%). Around 40% of the strains were resistant to the fourth -generation cephalosporins and 25% to aminoglicozides (Table 4) The lowest resistance rate was against colistin (8.1%). Almost a quarter of the *Pseudomonas* spp. strains were MDR (21/87) and 29.88% (26/87) were carbapenem-resistant.

#### 2.2.3. Multidrug Resistance (MDR)

Around 30% of all the pathogens were multidrug-resistant (MDR) (308/937). Although the number of MDR strains (186) increased in the post-pandemic period (2022–2023), compared to the number (124) registered during the pandemic (2021–2022), no significant difference between the percentage of MDR strains during and post-pandemic was observed.

Among the MDR Gram-positive pathogens, 31.80% of the *S. aureus* strains (76/239), 21.87% (14/64) of the *Enterococcus* spp., and 23.81% (10/42) of the *CoNS* strains were MDR.

**Figure 2:**
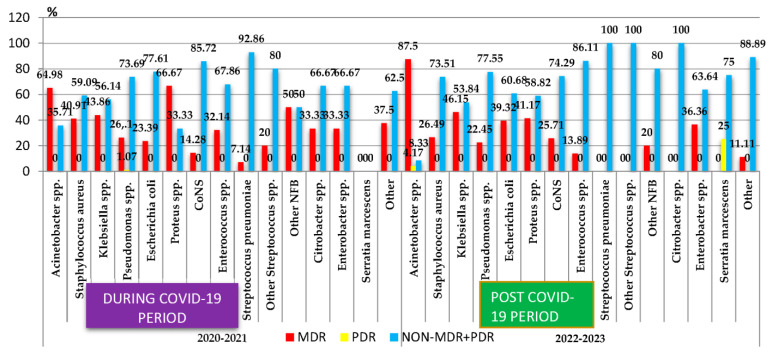
Distribution of the multidrug-resistant microorganisms isolated from samples from pediatric patients hospitalized in County Emergency Clinical Hospital Craiova, Romania, during and post-COVID-19. *NFB*—*Nonfermenting Gram-negative bacilli*; *CoNS*—*Coagulase-negative staphylococci*; MDR—Multidrug-Resistant; PDR—Pan-Drug-Resistant.

In the case of the most frequently isolated Gram-negative bacteria, a very high percentage of MDR isolates was found in the cases of *Acinetobacter* spp. (78.94%) (Figure 2). A high percentage of MDR strains was also highlighted in the case of other Gram-negative germs: *Klebsiella* spp. (45.18%) and *Proteus* spp. (50%). A quarter of the *Pseudomonas* spp. strains (21/87) and one-third of the *Escherichia coli* strains (61/184) and of the *Enterobacter* spp. strains (7/20) were MDR. Additionally, in 2022, two pan-drug-resistant (PDR) strains were isolated (*Acinetobacter* spp. and *Serratia marcescens*).

The comparative analysis of the percentage of MDR strains during the COVID-19 pandemic period (2020–2021) and post-pandemic period (2022–2023) showed a statistically significant increase post-pandemic in the case of the *Escherichia coli* strains (*p* < 0.05). The percentage of the MDR strains of *Klebsiella* spp. registered small variations in the two periods, while in the case of *Enterococcus* spp., it decreased almost three times. The percentage of MDR strains of *S. aureus*, *Proteus* spp., and *Pseudomonas* spp. also decreased, while that of *Acinetobacter* spp. MDR and *CoNS* MDR increased. However, these changes were not statistically significant (Figure 2).

#### 2.2.4. Carbapenem Resistance

**Figure 3:**
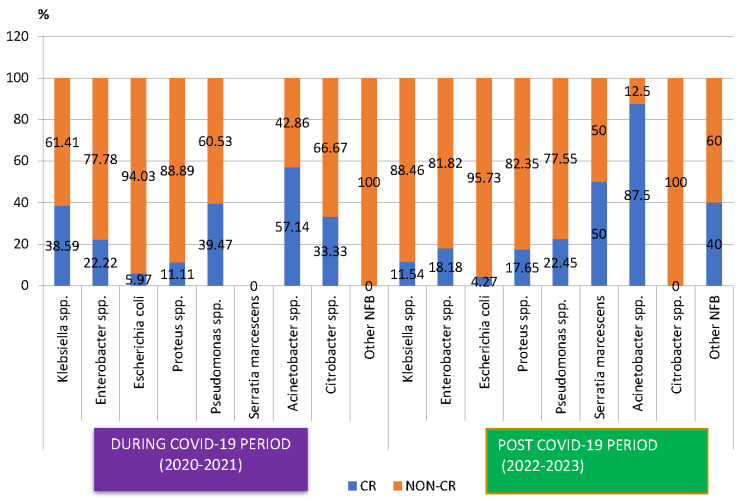
Distribution of carbapenem-resistant microorganisms isolated from samples from pediatric patients hospitalized in County Emergency Clinical Hospital Craiova, Romania, during and post-COVID-19. CR—carbapenem-resistant.

The proportion of carbapenem-resistant Gram-negative pathogens decreased, from 26.11% during the COVID-19 period to 17.97% in the post-COVID-19 period (Figure 3), with the difference being statistically significant (*p* < 0.05). The analysis of the trend for each pathogen showed that the percentage of carbapenem-resistant *Klebsiella* spp. strains decreased three times (*p* < 0.05). An important decrease was also recorded in the case of *Pseudomonas* spp. However, this was not significant. On the other hand, the percentage of carbapenem-resistant *Proteus* spp. and *Acinetobacter* spp. strains increased in the post-COVID-19 period, with the difference not being significant.

## 3. Discussion

Antimicrobial resistance is a globally recognized public health problem, with MDR infections having major consequences on morbidity and mortality in infected patients. Improper and unjustified administration of antibiotics in some cases during the COVID-19 pandemic led to the selection of MDR strains, which are also involved in most healthcare-associated infections. In this context, special attention should be paid to children, who represent a category of patients who are vulnerable due to their immature immune system and who require the administration of age-appropriate and dose-adjusted antibiotics, taking into account possible adverse effects.

According to the European Antimicrobial Resistance Surveillance Network (EARS-Net) Report for 2022, the AMR percentages remain a concern in the EU/EEA. The continuous increases in the percentages of carbapenem-resistant strains of *K. pneumoniae* and the vancomycin-resistant *E. faecium* are worrying elements [4].

The highest AMR percentages and estimated incidence of bloodstream infections with resistant bacteria were reported by countries in the south and east of Europe, with Romania being one of them [4]. Romania is in second place in the EU/EEA regarding the use of antibiotics, with a much higher risk of selecting MDR/XDR bacteria (55.1%) than the European average (38.6%) [12].

This situation can be explained by the inappropriate and excessive administration in the hospitals of antibiotics, including reserved antibiotics, the administration of antibiotics in viral infections of children without the indication of the specialist, the insufficient awareness by medical personnel of the risk of selecting multidrug-resistant strains, and the inadequate surveillance of AMR at local level.

A problem of great concern is the selection of multidrug-resistant microorganisms in the case of pediatric patients, because in Europe almost a third of infections with such pathogens are diagnosed in children [8].

The fact that certain antibiotics, like fluoroquinolones or tetracyclines, although effective against isolated pathogens, remain largely unused in pediatric patients due to safety concerns, with others having precise contraindications for child administration [8], limits the therapeutic possibilities in the case of infections with MDR microorganisms.

For these reasons, it is very helpful to identify early trends in AMR growth in children, so that urgent action can be taken to prevent their unfavorable consequences.

Little research has been published on antibiotic resistance of microorganisms involved in pediatric infections, but it has not focused on analyzing the consequences of the COVID-19 pandemic on antimicrobial resistance in children, especially in Romania. Only one study investigated the AMR rates of the main isolated bacterial pathogens in children, in 2022, after the COVID-19 pandemic, but without analyzing possible changes in the resistance due to the COVID-19 pandemic. According to this study, *Escherichia coli* was the most frequently isolated among Gram-negative microorganisms, and *Staphylococcus aureus* among Gram-positive bacteria microorganisms [13], consistent with our results.

In another retrospective cross-sectional study conducted at a tertiary-level university hospital, Western Saudi Arabia, the most commonly isolated organisms were *Klebsiella pneumoniae*, *Coagulase negative staphylococci (CONS)*, and *MRSA*, with almost 13% being MDR [14].

Since the beginning of the COVID-19 pandemic, an increase in multidrug-resistant pathogens has been observed. This can be explained by the administration of antibiotics to COVID-19 patients for prophylactic purposes or for the treatment of co-infections and secondary infections caused by bacteria. Approximately 75% of COVID-19 patients received antibiotics, while confirmed bacterial co-infection rates were less than 10% [15], leading to the selection of multidrug-resistant strains. The overuse of antibiotics during the pandemic mainly affected antimicrobial resistance of Gram-negative pathogens [16]. In our hospital, during the COVID-19 pandemic, COVID-19 patients admitted to the ICU received, prophylactically or for secondary co-infections, third-generation cephalosporins and quinolones, which may contribute to the selection of MDR pathogens.

A study conducted in Egypt showed an incidence of colonization of MDR bacteria among all patients admitted to the pediatric intensive care of approximately 9% [17].

A high frequency of MDR Gram-negative bacteria-related bacteremia in children was also reported in research conducted in Iran [18].

In our study, around 30% of all the pathogens were multidrug-resistant (MDR) (308/937). Although we found an increase in the number of MDR strains in the post-pandemic period (2022–2023) compared to the pandemic period (2021–2022), the difference was not statistically significant.

The majority (almost 55%) of the isolates were Gram-negative bacteria. This fact supports the finding that this type of infection has now made its way to infants and children [19]. These pathogens are also frequently implicated in the etiology of healthcare-associated infections in European acute care hospitals [20].

*Escherichia coli* was the most frequently isolated bacterium, with the highest resistance rate to amoxicillin/clavulanic acid and second-generation cephalosporins. One-third of the *Escherichia coli* strains were resistant to colistin. Over 30% of the strains were MDR, with a significant increase in the percentage post-pandemic (*p* < 0.05). Almost 5% of all the isolated strains were carbapenem-resistant.

According to the ECDC Annual Epidemiological Report, carbapenem resistance of the *Escherichia coli* invasive isolates was 0.7–0.4% in 2020–2021 [21].

Our research revealed that approximately 60% of *Klebsiella* spp. strains were isolated in the post-COVID-19 period. Almost half of the strains were MDR and approximately a third were carbapenem-resistant. These findings were consistent with the data registered at the national level, according to which the percentage of the carbapenem-resistant *Klebsiella pneumoniae* exceeds the European average by 4.75 times [12]. However, in the post-COVID-19 period, the percentage of carbapenem-resistant *Klebsiella* spp. strains has registered a significant decrease (*p* < 0.05). This may be due to the fact that not all the strains are tested for carbapenem resistance and to the specific pathology of the pediatric patients hospitalized in the two periods.

More than 70% of the strains were resistant to first- and second-generation cephalosporins and around 60% to third- and fourth-generation cephalosporins. The results were consistent with other studies [13,22,23,24,25,26,27] and correlate with the trend recorded at the national level. Thus, the reported antibiotic resistance levels for Romania indicated that, in 2020, *Klebsiella pneumoniae* had the highest resistance levels in third-generation cephalosporins, fluoroquinolones, and aminoglycosides [28]. According to the data published in 2023 in the ECDC Annual Epidemiological Report for 2021 [21], the percentage of invasive *Klebsiella pneumoniae* isolates resistant to third-generation cephalosporins (cefotaxime/ceftriaxone/ceftazidime) increased from 67.9% in 2020 to 70.8% in 2021 in Romania. Our research also revealed that a quarter of the *Klebsiella* spp. strains were resistant to colistin, which is a cause for great concern, as it is a backup antibiotic used in the case of carbapenem-resistant Gram-negative organisms [29].

More than 50% of the *Pseudomonas* spp. strains were isolated in our hospital in the post-COVID-19 period (56.32%), Almost 25% of the strains were MDR and approximately 20% were carbapenem-resistant, but a declining trend was recorded in the post-COVID-19 period compared to the pandemic period, although this was not significant. These values were much higher than those reported by the Chinese Infectious Disease Surveillance of Pediatrics (ISPED) program [30]. According to this, the detection rate of MDR pathogens in pediatric patients in 2021 was lower than in previous years, and the proportions of carbapenem-resistant *Pseudomonas aeruginosa* showed a decreasing trend in the past 6 years, reaching 6.7% in 2021. Additionally, the resistance rates found in our study were higher than those identified in another study conducted in pediatric patients in Romania [13,31] and in Iran. These findings can be explained by the selection of MDR strains due to the inappropriate and excessive administration of antibiotics in children, as well as by contracting healthcare-associated infections, with *Pseudomonas aeruginosa* remaining one of the major causes of healthcare-associated infection in Europe [4].

A very high percentage of MDR isolates was found in the case of all the isolated *Acinetobacter* spp. strains, with an important, but not statistically significant, increase in the post-pandemic period, the period in which a PDR strain was identified. These findings are consistent with the reported values at the national level. Those values show increasing extended resistance for *A. baumannii*, with a carbapenem resistance rate of 93.6% and multidrug-resistance rate of 89.4%, occupying third place for both indicators among EARS Net states [32]. The results are consistent with those from other studies [33,34,35,36]. Post-COVID-19, the percentage of carbapenem-resistant strains has increased compared to the pandemic period, but not significantly.

The highest percentage of the MDR Gram-positive pathogens in the entire studied period was registered in the case of *S. aureus* strains (31.80%). The recorded value was lower in the post-COVID-19 period, but without statistical significance. The highest resistance was recorded to penicillin, erytromycin, clarithromycin, and clindamycin. Approximately half of the tested strains were MRSA. The results were similar to those published by other researchers [13,37]. Although screening patients for multidrug-resistant pathogens and decolonization with intranasal mupirocin, together with chlorhexidine baths, is recommended to prevent MRSA infection, this can lead to resistance to mupirocin and chlorhexidine [8].

Over 20% of the *CoNS* strains isolated in our hospital in children between 2020 and 2023 were MDR, with an important increase in the post-COVID-19 period. Although the number of isolated strains is small (23), it is of particular importance because *CoNS* were the most common isolated pathogen from blood samples (44.23%).

In the case of *Enterococcus* spp., the percentage of MDR strains decreased almost three times in the post-COVID-19 period, given the fact that during this period two-thirds of the total strains were isolated. In another study [38], the authors also highlighted that Gram-positive bacteria have shown less global antimicrobial resistance (AMR) occurrence during and after the COVID-19 pandemic than Gram-negative bacteria. No association between COVID-19 and the incidence of carbapenem- or multidrug-resistant *Acinetobacter* spp. was found in this study.

Other researchers investigated the possible relationship between COVID-19 and increased AMR but, for comparison, most of them have taken into account data from the periods before and during the pandemic.

A study conducted in Romania by Mares et al., which investigated possible changes in uropathogen resistance in female patients before (1 September 2018–28 February 2019) and during the COVID-19 pandemic (1 September 2020–28 February 2021), found a significant increase in resistance to carbapenems [39].

Another study carried out in a tertiary care hospital in North India showed a significant increase in the carbapenem resistance rates for *Pseudomonas aeruginosa* and *Klebsiella pneumoniae* during the COVID period compared to pre-COVID period [40].

According to the ECDC Annual Epidemiological Report [21], carbapenem resistance was more frequently reported in *K. pneumoniae* than in *E. coli*. In 2021, low percentages were reported in northern and western parts of the WHO European Region. Romania was one of the 8 countries out of 45 that reported AMR percentages equal to or above 50%.

The main factors that can lead to the increase in antimicrobial resistance and the selection of MDR strains are the inadequate use of antibiotics without a positive microbiological result; the lack of continuity in the administration of antibiotics, either due to limited access or lack of knowledge; and insufficient application of the infection and disease prevention and control measures in hospitals. The screening of patients for MDR microorganisms, the isolation of those colonized/infected, the judicious prescription of antibiotics, the use of diagnostic procedures that are, as much as possible, less invasive and better accepted by patients [41], and high standards of infection prevention and control in the community and hospitals are important measures that can be adopted to comply with the Council of the EU Recommendation regarding the three AMR targets to be achieved by the EU by 2030 [1]. For Romania, these include reducing the total incidence of bloodstream infections with MRSA by 18%, and the third-generation cephalosporin-resistant *E. coli* by 5% and carbapenem-resistant *K. pneumoniae* by 5%, by 2030 against the baseline year 2019. Improving access to healthcare to establish an early and correct etiological diagnosis, limiting the empirical administration of antibiotics in the absence of microbiological expertise, using evidence-based guidelines, and rigorous maintenance of hospital hygiene are some measures that can contribute to preventing the spread of multidrug-resistant bacteria in pediatrics.

### Study Limitations

Our study has several limitations due to the fact that it is a retrospective study conducted in a single hospital, which may limit the generalizability of the findings to other settings. However, the hospital provides specialized medical assistance for children in the South-West Region of Romania, including very highly complex surgeries for newborns. The research we conducted is the only study that provides data on changes in antimicrobial resistance in the post-pandemic period in hospitalized pediatric patients, and is an important starting point for future research that will assess the impact of the COVID-19 pandemic on AMR in pediatric patients in Romania.

## 4. Materials and Methods

This research is a retrospective analysis of all the isolated bacterial pathogens collected from pediatric patients admitted to the Emergency Clinical County Hospital of Craiova, Romania, during and post-COVID-19 (January 2020-December 2023). The samples were collected from children <18 years old who were admitted to three pediatric wards of the tertiary teaching hospital: Pediatric Intensive Care Unit (PICU), Surgery, and Pediatrics.

Data were collected from the clinical pathology databases of the hospital including samples collected from patients with suggestive symptoms of infection. Samples collected according to the hospital procedure of patient screening were not included. The samples of blood, urine, sputum/tracheal aspirate (respiratory secretion), pus/wound swabs, exudates, intravascular catheters, and cerebrospinal fluid were processed at the Hospital’s Laboratory of Microbiology.

Specialized bottles provided with the Bact/Alert 70^®^ 3D (Mediclim SRL, Bucharest, Romania) automated system were used to collect blood samples. For each patient, a set of two culture bottles was collected, including one bottle for aerobic bacteria and one for anaerobic bacteria.

The retrospective analysis included all positive bacterial cultures from pediatric patients admitted to selected wards in the studied period. The exclusion criteria of the samples were as follows: bacterial duplicates, defined as the same pathogen with the same resistance profile isolated from the same patient and the same site of infection; and samples collected less than 30 days apart, during which the same pathogen was isolated. Multidrug resistance was analyzed by taking into account the resistance to at least three different antibiotic groups: aminoglycosides, cephalosporins, carbapenems, tetracyclines, and fluoroquinolones [42].

An isolated pathogen was classified as a multidrug-resistant (MDR) pathogen if acquired non-susceptibility was demonstrated to at least one agent in three or more antimicrobial categories [42].

If the isolated pathogen was non-susceptible to all agents tested in the hospital, it was considered pan-drug-resistant (PDR) [43].

The Vitek 2 Compact system was used for the identification of the isolated strains and the analysis of the resistance patterns for the action of the appropriate antibiotics [44].

The antibiotic susceptibility test was performed following the guidelines provided by the Clinical Laboratory Standard Institute (CLSI) [45].

Information about patients’ age, sex, hospital department, sample type, site of infection, and antimicrobial resistance pattern were stored in the Hospital‘s Information System. All the obtained data were entered into Microsoft Excel(Microsoft Office 365). Continuous variables like age are expressed as mean ± STDEV (standard deviation). The distribution of the microorganisms was analyzed and is expressed as percentages. The percentage of resistant strains was calculated by dividing the resistance strains by the tested strains. To assess the impact of the COVID-19 pandemic on multidrug resistance, the study period was divided into two periods: during the COVID-19 period (2020–2021) and the post-COVID-19 period (2022–2023). The statistical evaluation of possible differences in antibiotic resistance between the COVID-19 period and the post-COVID period was performed individually for each pathogen, using the chi square test for independence, or the Fisher exact test for small groups. Epi Info software, version 7.2.4.0., was used for all statistical analyses. A two-sided *p*-value ≤ 0.05 was considered to be statistically significant.

## 5. Conclusions

Our findings revealed the increase in the post-COVID-19 period of the prevalence of MDR strains of *Acinetobacter* spp., *CoNS*, and *Escherichia coli* (in this case, the increase was significant) isolated in pediatric patients’ samples. This can lead to an increased risk of unfavorable evolution and death, given that it is a category of patients in whom the administration of certain antibiotics is contraindicated. A significant decline in the trend of the carbapenem-resistant Gram-negative pathogens identified in our research was shown in the post-COVID-19 pandemic, especially in the case of *Klebsiella* spp. strains, which can be explained by the fact that not all the strains are tested for carbapenem resistance. Additionally, during the COVID-19 pandemic, due to the limited access at the level of our hospital, particularly severe pediatric cases were treated, with or without co-infection with SARS-CoV-2. We believe that future extensive studies are needed to evaluate the possible consequences of the COVID-19 pandemic on AMR, so that the necessary measures can be taken.

## Figures and Tables

**Table 1:** Distribution of samples from pediatric patients hospitalized in County Emergency Clinical Hospital Craiova, Romania, between 2020 and 2023.

	Year				
Specimen Type	2020	2021	2022	2023	Total
Central venous catheter	4 (1.50%)	3 (1.27%)	1 (0.65%)	-	8 (0.95%)
Nose/pharynx	20 (7.52%)	20 (8.48%)	9 (5.85%)	16 (8.47%)	65 (7.69%)
Blood	13 (4.89%)	9 (3.81%)	11 (7.14%)	8 (4.23%)	41 (4.85%)
Pus/wound swabs	87 (32.71%)	61 (25.85%)	22 (14.28%)	67 (35.45%)	237 (28.05%)
Urine	64 (24.06%)	64 (27.12%)	40 (25.98%)	37 (19.57%)	205 (24.26%)
Cerebrospinal fluid	1 (0.37%)	-	-	1 (0.53%)	2 (0.23%)
Respiratory tract	54 (20.30%)	63 (26.69%)	61 (39.61%)	53 (28.04%)	231 (27.34%)
Other	23 (8.65%)	16 (6.78%)	10 (6.49%)	7 (3.71%)	56 (6.63%)
TOTAL	266	236	154	189	845

**Table 2:** Distribution of the isolated pathogens during and post-COVID-19.

Species	During COVID-19 (2020–2021)		Post-COVID-19 (2022–2023)		Total
	No.	%	No.	%	No
*Klebsiella* spp.	57	42.22	78	57.78	135
*Escherichia coli*	67	36.41	117	63.59	184
*Pseudomonas* spp.	38	43.68	49	56.32	87
*Enterococcus* spp.	28	43.75	36	56.25	64
*Staphylococcus aureus*	88	36.82	151	63.18	239
*Acinetobacter* spp.	14	36.84	24	63.16	38
Other NFB *	6	54.54	5	45.46	11
*Enterobacter* spp.	9	45	11	55	20
*Proteus* spp.	9	36.42	17	65.38	26
*Coagulase-negative Staphylococci*	7	16.67	35	83.33	42
*Streptococcus pneumoniae*	28	50.91	27	49.09	55
*Other Streptococcus* spp.	5	45.46	6	54.54	11
*Citrobacter* spp.	3	75	1	25	4
*Serratia marcescens*	0	0	4	100	4
Other	8	47.06	9	52.94	17
Total	367	39.17	570	60.83	937

* Other *NFB*—Other *Nonfermenting Gram-negative bacilli*.

**Table 3:** Antimicrobial resistance pattern of the main Gram-positive pathogens isolated from pediatric patients hospitalized in the County Emergency Clinical Hospital Craiova, Romania, 2020–2023.

Antimicrobial Agent	*Staphylococcus aureus* (n = 239)	*Streptococcus* *pneumoniae* (n = 55)	*Enterococcus* spp. (n = 64)
Ciprofloxacin	59/219 (26.94%)	-	32/61 (52.46%)
Clindamycin	161/235 (68.51%)	7/50 (14%)	-
Clarithromycin	153/233 (65.66%)	14/49 (28.57%)	-
Doxycycline	57/225 (25.33%)	10/51 (19.61%)	23/52 (44.23%)
Erythromycin	179/237 (5.53%)	16/49 (32.65%)	-
Linezolid	0/219 (0%)	0/39 (0%)	2/54 (3.70%)
Penicillin	190/237 (80.17%)	32/42 (76.19%)	25/46 (54.34%)
Rifampicin	252/174 (53.16%)	0/40 (0%)	-
Tetracycline	63/186 (33.87%)	14/33 (42.42%)	-
Oxacillin	114/175 (46.34%)	-	-
Vancomycin	2/22 (9.09%)	0/48 (0%)	4/48 (8.33%)
Teicoplanin	16/66 (24.24%)	-	8/30 (26.67%)
Gentamicin	69/206 (33.49%)	-	-
Levofloxacin	24/162 (14.28%)	8/37 (21.62%)	7/24 (29.17%)
Moxifloxacin	35/200 (17.5%)	3/48 (6.25%)	-

Percentage of each column is calculated by dividing the resistance strains to the tested strains; ‘-’ not tested.

**Table 4:** Antimicrobial resistance pattern of the main Gram-negative pathogens isolated from pediatric patients hospitalized in the County Emergency Clinical Hospital Craiova, Romania, 2020–2023.

Antimicrobial Agent	*Escherichia coli* (n = 184)	*Klebsiella* spp. (n = 135)	*Pseudomonas* spp. (n = 87)
Amoxicillin/ clavulanic acid	100/161 (62.11%)	80/115 (69.56%)	-
Ceftazidime	68/161 (42.33%)	73/120 (60.83%)	35/81 (43.21%)
Ceftriaxone	47/146 (35.58%)	68/114 (59.65%)	10/14 (71.43%)
Cefazolin	52/122 (42.63%)	56/79 (70.88%)	-
Cefuroxime	63/102 (61.76%)	49/67 (73.13%)	-
Cefepime	37/116 (31.89%)	39/74 (52.70%)	23/59 (38.98%)
Imipenem	7/139 (5.03%)	21/105 (20%)	23/70 (32.85%)
Meropenem	4/91 (4.39%)	14/67 (20.89%)	17/62 (27.42%)
Ciprofloxacin	51/158 (32.27%)	44/118 (37.28%)	13/79 (14.46%)
Levofloxacin	18/95 (18.94%)	12/65 (18.46%)	14/52 (26.93%)
Piperacillin/ tazobactam	42/89 (47.19%)	43/74 (58.11%)	18/64 (28.12%)
Colistin	34/129 (26.36%)	26 (24.76%)	6/74 (8.10%)
Gentamicin	50/147 (34.01%)	53/97 (54.64%)	18/75 (24%)
Amikacin	31/111 (27.93%)	28/71 (39.43%)	10/49 (20.41%)
Aztreonam	33/139 (23.74%)	42/105 (40%)	14/56 (25%)

Percentage of each column is calculated by dividing the resistance strains to the tested ones; ‘-’ not tested.

## Data Availability

Data are contained within the article.

## Supplementary Materials

The following supporting information can be downloaded at: https://www.mdpi.com/article/10.3390/antibiotics13100966/s1. Table S1: Distribution of the pathogens per year and department, County Emergency Clinical Hospital Craiova, Romania, 2020–2023. Table S2: Distribution of the microorganisms isolated from samples from pediatric patients, County Emergency Clinical Hospital Craiova, Romania, 2020–2023.
