# Supplementary material for: Antibiotic Resistance Pattern of Pathogens Isolated from Pediatric Patients during and after the COVID-19 Pandemic
Source: Antibiotics (Basel). 2024 Oct 13;13(10):966. doi: 10.3390/antibiotics13100966 (PMC11505055; doi:10.3390/antibiotics13100966)
Supplement: Supplementary file 1 [file antibiotics-13-00966-s001.zip › antibiotics-3236317-supplementary.pdf]

Table S1 Distribution of the pathogens per year and department, County Emergency Clinical Hospital Craiova, Romania, 2020-2023

**Bacterial species**		**2020**			**2021**			**2022**			**2023**			**TOTAL**
		**PICU**	**Surgery**	**Pediatrics**	**PICU**	**Surgery**	**Pediatrics**	**PICU**	**Surgery**	**Pediatrics**	**PICU**	**Surgery**	**Pediatrics**	
Klebsiella spp.	No.	15	3	16	5	1	17	1	5	26	12	13	21	135
	% within Bacteria	11.11	2.22	11.85	3.71	0.74	12.59	0.74	3.71	19.26	8.88	9.63	15.56	100
	% within Ward	23.44	4.28	25	9.81	3.57	18.89	2.08	8.07	19.55	12.25	15.85	14.28	14.41
Escherichia coli	No.	3	22	15	-	10	17	5	18	33	11	17	33	184
	% within Bacteria	1.63	11.96	8.15	-	5.44	9.24	2.72	9.78	17.93	5.98	9.24	17.93	100
	% within Ward	4.68	31.43	23.43	-	35.72	18.89	10.42	29.03	24.81	11.23	20.73	22.45	19.64
Pseudomonas spp.	No.	9	6	5	6	2	10	2	3	9	13	7	15	87
	% within Bacteria	10.34	6.90	5.75	6.90	2.30	11.49	2.30	3.45	10.34	14.95	8.04	17.24	100
	% within Ward	14.06	8.57	7.81	11.76	7.15	11.11	4.17	4.84	6.77	13.26	8.53	10.21	9.28
Enterococcus spp.	No.	1	6	2	4	6	9	7	1	6	6	6	10	64
	% within Bacteria	1.57	9.37	3.13	6.25	9.37	14.06	10.94	1.57	9.37	9.37	9.37	15.63	100
	% within Ward	1.57	8.57	3.13	7.84	21.43	10	14.58	1.61	4.51	6.12	7.32	6.81	6.83
Staphylococcus aureus	No.	16	19	12	17	5	19	13	27	30	25	22	34	239
	% within Bacteria	6.69	7.95	5.02	7.11	2.09	7.95	5.44	11.30	12.55	10.46	9.21	14.23	100
	% within Ward	25	27.14	18.75	33.33	17.85	21.11	27.08	43.55	22.55	25.51	26.83	23.13	25.51
Acinetobacter spp.	No.	7	1	1	3	1	1	5	2	5	9	1	2	38
	% within Bacteria	18.42	2.63	2.63	7.90	2.63	2.63	1.16	5.26	13.16	23.69	2.63	5.26	100
	% within Ward	10.93	1.43	1.57	5.88	3.57	1.11	10.42	3.23	3.76	9.18	1.22	1.36	4.06
*Other NFB**	No.	-	3	-	2	-	1	-	1	-	1	-	3	11
	% within Bacteria	-	27.27	-	18.18	-	9.09	-	9.09	-	9.09	-	27.27	100
	% within Ward	-	4.28	-	3.92	-	1.11	-	1.11	-	1.11	-	2.04	1.17
Enterobacter spp.	No.	1	2	3	1	1	1	-	-	3	3	2	3	20
	% within Bacteria	5	10	15	5	5	5	-	-	15	15	10	15	100
	% within Ward	1.57	2.86	4.68	1.96	3.57	1.11	-	-	2.26	3.06	2.44	2.04	2.14
Proteus spp.	No.	1	2	5	1	-	-	-	3	5	1	3	5	26
	% within Bacteria	3.85	7.68	19.23	3.85	-	-	-	11.54	19.23	3.85	11.54	19.23	100
	% within Ward	1.57	2.86	7.81	1.96	-	-	-	4.84	3.76	1.02	3.66	3.40	2.77
Coagulase-negative Staphylococci	No.	3	1	3	-	-	-	-	-	-	13	6	16	42
	% within Bacteria	7.15	2.38	7.15	-	-	-	-	-	-	30.95	14.28	38.09	100
	% within Ward	4.68	1.43	4.68	-	-	-	-	-	-	13.27	7.32	10.88	4.48

Bacterial species		2020			2021			2022			2023			TOTAL
		PICU	Surgery	Pediatrics	PICU	Surgery	Pediatrics	PICU	Surgery	Pediatrics	PICU	Surgery	Pediatrics	
Streptococcus pneumoniae	No.	8	1	1	6	-	12	11	-	10	3	-	3	55
	% within Bacteria	14.55	1.82	1.82	10.91	-	21.82	20	-	18.18	5.45	-	5.45	100
	% within Ward	12.5	1.43	1.57	11.76	-	13.33	22.92	-	7.52	3.06	-	2.04	5.87
Other Streptococcus spp.	No.	-	2	1	1	1	-	1	1	-	-	4	-	4
	% within Bacteria	-	18.18	9.09	9.09	9.09	-	9.09	9.09	-	-	36.37	-	100
	% within Ward	-	2.86	1.57	1.96	3.57	-	2.08	1.61	-	-	4.88	-	1.17
Citrobacter spp.	No.	-	2	-	1	-	-	-	-	-	-	1	-	4
	% within Bacteria	-	50	-	25	-	-	-	-	-	-	25	-	100
	% within Ward	-	2.86	-	1.96	-	-	-	-	-	-	1.22	-	0.43
Serratia marcescens	No.	-	-	-	-		-	1	-	-	1	-	2	4
	% within Bacteria	-	-	-	-	-	-	25	-	-	25	-	50	100
	% within Ward	-	-	-	-	-	-	2.08	-	-	1.02	-	1.36	0.43
Other	No.	-	-	-	4	1	3	2	1	6	-	-	-	17
	% within Bacteria	-	-	-	23.53	5.88	17.65	11.76	5.88	35.30	-	-	-	100
	% within Ward	-	-	-	7.84	3.57	3.34	4.17	1.61	4.51	-	-	-	1.81
**TOTAL**	No.	64	70	64	51	28	90	48	62	133	98	82	147	937
	% within Bacteria	6.83	7.43	6.83	5.44	2.99	9.61	5.12	6.62	14.19	10.46	8.75	15.69	100
	% within Ward	100	100	100	100	100	100	100	100	100	100	100	100	100

** Other NFB — Other Nonfermenting Gram-negative bacilli*

Table S2. Distribution of the microorganisms isolated from samples from pediatric patients, County Emergency Clinical Hospital Craiova, Romania, 2020-2023

**Bacterial species**		**Urine**	**Respiratory tract**	**Nose/pharynx**	**Blood**	**Central venous catheter**	**Pus/wound swabs**	**Cerebrospinal fluid**	**Other**	**TOTAL**
Klebsiella spp.	No.	62	28	14	1	-	23	-	7	135
	% within Bacteria	45.93	20.74	10.37	0.74	-	17.04	-	5.18	100
	% within specimen type**	28.44	11.62	12.96	1.92	-	17.04	-	12.5	14.41
Escherichia coli	No.	94	10	10	1	-	64	-	5	184
	% within Bacteria	51.09	5.43	5.43	0.55	-	34.78	-	2.72	100
	% within specimen type	43.12	4.15	9.26	1.42	-	25.50	-	8.93	19.64
Pseudomonas spp.	No.	8	38	11	1	1	21	-	7	87
	% within Bacteria	9.19	43.68	12.64	1.15	1.15	24.14	-	8.05	100
	% within specimen type	3.67	15.77	10.19	1.92	12.5	8.37	-	12.5	9.28
Enterococcus spp.	No.	26	12	-	3	-	18	-	5	64
	% within Bacteria	40.63	18.75	-	4.68	-	28.12	-	7.82	100
	% within specimen type	11.95	4.98	-	5.77	-	7.17	-	8.93	6.83
Staphylococcus aureus	No.	3	66	64	5	5	82	2	12	239
	% within Bacteria	1.25	27.62	26.78	2.09	2.09	34.31	0.84	5.02	100
	% within specimen type	1.37	27.39	59.26	9.62	62.5	32.67	66.67	21.43	25.51
Acinetobacter spp.	No.	-	22	2	4	1	7	-	2	38
	% within Bacteria	-	57.90	5.26	10.53	2.63	18.42	-	5.26	100
	% within specimen type	-	9.13	1.85	7.69	12.5	2.79	-	3.57	4.06
*Other NFB**	No.	3	3	-	-	-	4	-	1	11
	% within Bacteria	27.27	27.27	-	-	-	36.37	-	9.09	100
	% within specimen type	1.37	1.25	-	-	-	1.60	-	1.79	1.17
Enterobacter spp.	No.	7	4	4	1	-	4	-	-	20
	% within Bacteria	35	20	20	5	-	20	-	-	100
	% within specimen type	3.21	1.66	3.70	1.92	-	1.60	-	-	2.14
Proteus spp.	No.	13	5	1	-	-	7	-	-	26
	% within Bacteria	50	19.23	3.84	-	-	26.93	-	-	100
	% within specimen type	5.97	2.07	0.93	-	-	2.78	-	-	2.77
Coagulase-negative Staphylococci	No	-	1	-	23	1	8	-	9	42
	% within Bacteria	-	2.38	-	54.76	2.38	19.05	-	21.43	100
	% within specimen type	-	0.41	-	44.23	12.5	3.19	-	16.07	4.48

**Bacterial species**		**Urine**	**Respiratory tract**	**Nose/pharynx**	**Blood**	**Central venous catheter**	**Pus/wound swabs**	**Cerebrospinal fluid**	**Other**	**TOTAL**
Streptococcus pneumoniae	No.	-	50	-	-	-	1	1	3	55
	% within Bacteria	-	90.91	-	-	-	1.82	1.82	5.45	100
	% within specimen type	-	20.75	-	-	-	0.39	33.33	5.35	5.87
Other Streptococcus spp.	No.	-	1	-	-	-	9	-	1	11
	% within Bacteria	-	9.09	-	-	-	81.82	-	9.09	100
	% within specimen type	-	0.41	-	-	-	3.58	-	1.79	1.17
Citrobacter spp.	No.	1	1	-	-	-	2	-	-	4
	% within Bacteria	25	25	-	-	-	50	-	-	100
	% within specimen type	0.46	0.41	-	-	-	0.80	-	-	0.43
Serratia marcescens	No.	1	-	2	1	-	-	-	-	4
	% within Bacteria	25	-	50	25	-	-	-	-	100
	% within specimen type	0.46	-	1.85	1.92	-	-	-	-	0.43
Other	No.	-	-	-	12	-	1	-	4	17
	% within Bacteria	-	-	-	70.50	-	5.88	-	23.53	100
	% within specimen type	-	-	-	23.07	-	0.39	-	7.14	1.81
**TOTAL**	No.	218	241	108	52	8	251	3	55	937
	% within Bacteria	23.27	25.72	11.53	5.55	0.85	26.79	0.32	5.97	100
	% within specimen type	100	100	100	100	100	100	100	100	100

* *Other NFB— Other Nonfermenting Gram-negative bacilli;*

** *% within specimen type is calculated by dividing the number of specific strains found in a particular sample by all the pathogens found in the same sample.*
